# Cell-specific priors rescue differential gene expression in spatial spot-based technologies

**DOI:** 10.1093/bib/bbae621

**Published:** 2024-12-16

**Authors:** Ornit Nahman, Timothy J Few-Cooper, Shai S Shen-Orr

**Affiliations:** Department of Immunology, Rappaport Faculty of Medicine, Technion—Israel Institute of Technology, 1 Efron St., Haifa, 3525433, Israel; Department of Immunology, Rappaport Faculty of Medicine, Technion—Israel Institute of Technology, 1 Efron St., Haifa, 3525433, Israel; Department of Immunology, Rappaport Faculty of Medicine, Technion—Israel Institute of Technology, 1 Efron St., Haifa, 3525433, Israel

**Keywords:** spatial transcriptomics, differentially expressed genes, gene specificity, deconvolution

## Abstract

Spatial transcriptomics (ST), a breakthrough technology, captures the complex structure and state of tissues through the spatial profiling of gene expression. A variety of ST technologies have now emerged, most prominently spot-based platforms such as Visium. Despite the widespread use of ST and its distinct data characteristics, the vast majority of studies continue to analyze ST data using algorithms originally designed for older technologies such as single-cell (SC) and bulk RNA-seq—particularly when identifying differentially expressed genes (DEGs). However, it remains unclear whether these algorithms are still valid or appropriate for ST data. Therefore, here, we sought to characterize the performance of these methods by constructing an *in silico* simulator of ST data with a controllable and known DEG ground truth. Surprisingly, our findings reveal little variation in the performance of classic DEG algorithms—all of which fail to accurately recapture known DEGs to significant levels. We further demonstrate that cellular heterogeneity within spots is a primary cause of this poor performance and propose a simple gene-selection scheme, based on prior knowledge of cell-type specificity, to overcome this. Notably, our approach outperforms existing data-driven methods designed specifically for ST data and offers improved DEG recovery and reliability rates. In summary, our work details a conceptual framework that can be used upstream, agnostically, of any DEG algorithm to improve the accuracy of ST analysis and any downstream findings.

## Introduction

In recent years, spatial transcriptomics (ST) has gained immense popularity for its ability to spatially resolve gene expression in the context of 2D tissue structure. In particular, spot-based technologies (e.g. 10X Visium) have been widely adopted owing to relatively low costs and user-friendly setup. Despite the merits of spot-based approaches, these platforms present distinct technological challenges, including: (i) spot-level resolution, which ranges from capturing a few to hundreds of cells per spot and which can create significant levels of spot heterogeneity; (ii) diffusion (“leakage”) of transcripts into neighboring spots; and (iii) akin to, yet worse than, single-cell (SC) RNA-seq data—sparsity and low sequencing depth [1–3]. Regardless of these challenges, this technology remains an invaluable tool for biomedical research. Of these three main challenges, spot heterogeneity has received the most attention, with a plethora of deconvolution algorithms developed in an attempt to mitigate the issue [4–11]. However, even with deconvolution, issues arising from spot heterogeneity may not yet be fully resolved.

A common analysis in the transcriptomic field is the identification of differentially expressed genes (DEGs). In ST data, one approach to DEG detection seeks to identify spatially variable genes (SVGs), whereby coherent patterns of expression are searched for in space. SVGs are typically used to define clusters or niches within the tissue, in an unsupervised manner. In contrast, a more classical, supervised approach, which is widely used, predefines regions of interest based on the origin of the tissue sample or by morphological examination of accompanying Hematoxylin and Eosin (H&E) images and computes DEGs between pairs of selected areas. While several novel, ST-specific algorithms exist for SVG-based DEG analysis [12–16], the algorithms used in this latter scenario are, in many cases, designed for older technologies (e.g. bulk RNA-seq, scRNA-seq) [17–19]. Given the unique characteristics and challenges of ST data, the efficacy of such DEG algorithms in accurately recovering DEGs in this manner is unclear. Indeed, a number of common DEG methods were recently shown to exhibit inflated error rates when spatial dependencies are present in the data [20], emphasizing the need to explore this further. Moreover, despite the recent emergence of ST-specific DEG algorithms [21, 22], these methods remain under-utilized and under-tested. We therefore chose to focus our attention on this type of supervised ST DEG analysis.

Assessing the accuracy of DEG algorithms on ST data is challenging owing to a lack of knowable, ground truth. Thus, we evaluated the performance of both classic and ST-specific DEG algorithms—between two regions and/or conditions—by constructing and using a novel simulation platform capable of generating simulated tissues with known DEGs. Notably, we demonstrate that all methods display relatively poor performance, and we further identify the technology-specific factors responsible for this. Finally, we propose a conceptual framework to enhance the reliability of ST DEG results by integrating prior knowledge of cell type–specific expression.

## Results

### Classic algorithms for differentially expressed gene analysis perform poorly on realistic, simulated spatial data

**Figure 1:**
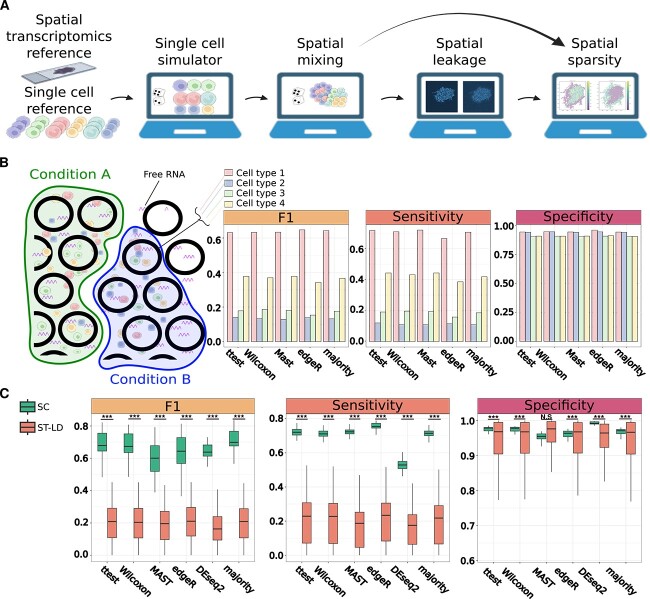
A novel spatial simulation pipeline highlights the poor performance of classic DEG algorithms on spatial data. (A) A novel simulation pipeline to simulate spatial tissues with known DEGs. In brief, the simulation platform: (i) utilizes SC and 10X Visium ST references as input; (ii) simulates SC data consisting of two conditions, with known DEGs; (iii) allocates SCs to spots in two-dimensional space, according to user-controlled simulation parameters; and (iv) optionally, simulates leakage of transcripts to neighboring spots and downsampling of transcripts to emulate sparsity. (B) A DEG detection scheme and a set of evaluation metrics allow comparisons between different DEG methods. Left: Each simulated tissue is divided into two regions in accordance with the simulated conditions. In each spot, multiple different cell types can reside—generating the cellular heterogeneity characteristic of spot-based ST technologies and which is referred to here as “mixing.” Each region exclusively contains cells from one condition, thus differential expression is computed between each cell type of each region. It is important to note that in spots lacking cells, transcripts may still be present due to transcript leakage. Right: A hypothetical example of the evaluation metrics used—namely, F1 (harmonic mean of precision and recall), sensitivity, and specificity, which are calculated independently for each DEG method and per cell type. (C) The ability to accurately detect DEGs in realistic spatial simulations is significantly diminished relative to SC data. F1, sensitivity, and specificity scores derived from either simulated SC data or ST-LD simulated data, stratified by each DEG detection algorithm tested. F1 scores of the different DEG methods for ST-LD are not significantly different (Kruskal–Wallis *P*-value = 0.1). (SC, single cell; ST-LD, ST with leakage and downsampling) (^*^P ≤ .05, ^*^^*^P ≤ .01, ^*^^*^^*^P ≤ .001).

To assess the performance and accuracy of DEG algorithms on ST data, we established a simulation pipeline capable of generating spatial data *in silico* that closely resembles real-world data, but that also includes a well-defined ground truth of DEGs. In brief, our first-of-its-kind simulation scheme combines simulated single cells (SCs), with known DEGs, into spots distributed across a 2D tissue slice, with optional inclusion of simulated transcript leakage to neighboring spots, and optional downsampling of the transcripts within each spot to mimic the sparsity and depth of real ST data (see Methods) (Fig. 1A). Moreover, we incorporated unique aspects of ST data, such as the density of cells per spot and uniformity of cell type occurrence across spots, into our simulation scheme (see Methods) (Supplementary Fig. 1A and B) and provide tunable parameters (see Supplementary Table 1) for DEG generation in order to collectively investigate a wide range of different scenarios. In total, we conducted 800 simulations across 160 distinct parameter configurations to not only comprehensively appraise DEG algorithms but also identify spatial-specific parameters affecting their performance. Our evaluation compared two distinct biological conditions, present on the same simulated tissue slice, that exhibit known and controllable differences in gene expression per cell type. Each spot that contains a specific cell type participates in the evaluation of algorithm accuracy, assessed through F1, sensitivity, and specificity metrics, for that particular cell type (see Methods) (Fig. 1B).

We initially focused our efforts on the simulation configuration that best recaptures the characteristics and dynamic range of real ST data—as learned and inferred from nine different spatial datasets (Supplementary Fig. 2)—which incorporates both leakage and downsampling effects (hereafter referred to as “ST-LD”). By using ST-LD, we tested common, “classic” DEG algorithms, that is, algorithms oblivious to the distinct nature of ST data and that predate spatial methods (see Methods), contrasting performance here to direct analysis of the same data in SC space.

In all cases, data were preprocessed according to common practices (see: Methods) and normalized using *SCTransform*—a method commonly applied to both SC and ST data—where applicable. Strikingly, we observed a significant and substantial decrease in F1 and sensitivity scores in comparison to the analysis of SC data (*t*-test corrected with Bonferroni, *P* < 9e-10) (Fig. 1C). While a decrease was expected due to the lack of ST-specific optimizations in these DEG algorithms, test scores were remarkably low (mean F1 scores = 0.2 ± 0.014) and moreover, similar across all DEG methods tested, including when we combined the results of each DEG algorithm through majority voting (Kruskal–Wallis *P* = .1) (Fig. 1C). Interestingly, while spatial characteristics, including the density of cells in a spot and the uniformity of cells in space, do affect these results, the effects are consistent across all DEG methods tested and even favorable spatial parameters failed to rescue DEG recovery to any appreciable, practical level (Supplementary Fig. 3). Such results suggest that the low performance of classic DEG algorithms may be attributable to an inherent property of spot-based ST data or, alternatively, the preprocessing of ST data, as opposed to a failure of the DEG methods themselves.

### Distinct normalization schemes are unable to rescue differentially expressed genes in spatial transcriptomics data

**Figure 2:**
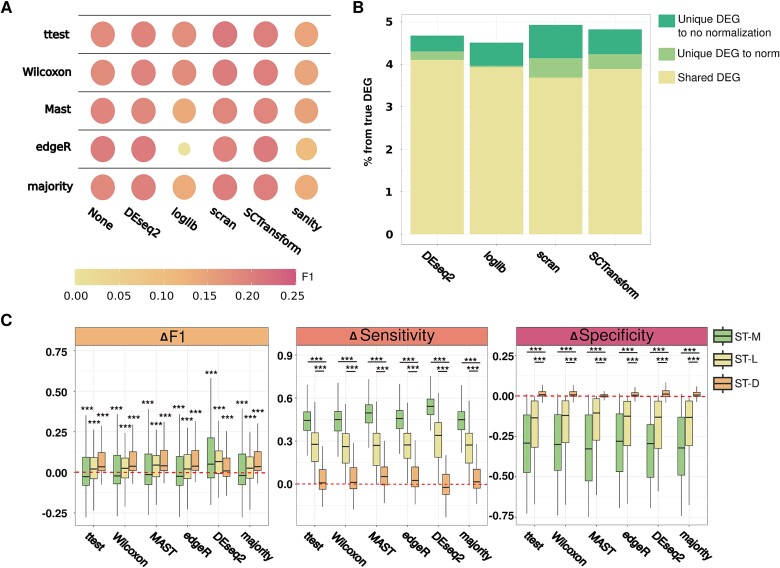
Normalization methods have a negligible impact on DEG recovery in spatial data. (A) DEG detection in ST data is not rescued by different normalization methods. A dot plot showing the F1 scores of all combinations of DEG algorithms and normalization techniques tested. Note that the range of F1 scores obtained resides between 0 and 0.25, while the theoretical F1 range is 0–1. For most combinations, only small practical differences are noted yet these remain significant (Kruskal–Wallis for each DEG separately, *P*-values <2e-16). (B) The percentage of both unique and true DEGs detected by utilizing different normalization algorithms is limited. In brief, the detection of true DEGs, as a percentage of total DEGs, was assessed by a *t*-test per normalization method and compared to unnormalized data. DEGs detected by both normalized and unnormalized data, and DEGs detected by either method alone are shown. While the majority of true DEGs detected are shared, unnormalized data yielded a marginally higher percentage of unique DEGs; however, the percentage of DEGs accurately detected overall, in all cases, remains very low (~5%). (C) Spot heterogeneity is, alone, sufficient to hinder the accurate identification of DEGs. F1, sensitivity, and specificity scores derived from various combinations of unique ST configurations that isolate different characteristics of spatial data, and which are expressed as differences (Δ) relative to the baseline analysis of ST-LD (see Fig. 1C). Scores above zero represent methods with improved, relative performance and vice versa (ST-M, ST with only mixing; ST-L, ST with leakage; ST-D, ST with downsampling) (^*^P ≤ .05, ^*^^*^P ≤ .01, ^*^^*^^*^P ≤ .001).

Given the unexpectedly low performance of all tested DEG algorithms on ST data, we next sought to pinpoint the factors responsible for this. As normalization methods can have a significant impact on DEG results [23], we hypothesized that by employing different normalization methods we may yield diverse and/or improved DEG results. However, we observed minimal variation among the different normalization algorithms, closely resembling the variation observed between DEG algorithms (Fig. 2A). While F1 scores were significantly different between normalization methods when tested for each DEG algorithm separately (Kruskal–Wallis *P*-value <2e-16), the differences were ultimately without practical value as they all remain poor overall (F1 ≈ 0.2).

Subsequently, to test whether normalization itself reduces the ability to accurately call DEGs, we compared unique DEGs, called per normalization method, to an unnormalized case. Surprisingly, we observed a higher amount of true, unique DEGs in the unnormalized approach (Fig. 2B); however, the difference is marginal, and, in all cases, only ~4%–5% of the true DEGs are correctly identified. Collectively, these findings suggest that while normalization methods may differ statistically and mathematically, their practical impact on DEG analysis in ST data is minimal and normalization methods commonly used for ST data are unable to rescue the poor recovery of true DEGs.

### Spot-level heterogeneity is primarily responsible for the decrease in accurate differentially expressed gene recovery

Since we observed similar results across varying DEG and normalization methods, we hypothesized that the underlying cause of inaccurate DEG recovery may be more datatype-inherent in nature. Consequently, we turned our attention to assessing the contribution of each unique spatial characteristic to the observed lack of performance. By utilizing our simulation pipeline, we generated models from intermediate stages that incorporate various combinations of these characteristics. Each simulation comprised a mixture of cells in spots (“ST-M”), which represents the spatial and cellular heterogeneity of ST data—an unavoidable property of spot-based technologies—and could additionally include, in isolation from one another, leakage of transcripts (“ST-L”), downsampling of transcripts per spot (“ST-D”), or both (“ST-LD,” as used in Fig. 1) (Supp Fig. 1C). While ST-M, ST-L, and ST-D configurations are unrealistic scenarios, unachievable with current technology, they serve as tools to help us dissect the problem. We used ST-LD, the most realistic configuration (see: Supp Fig. 2), as a baseline for comparison against all other configurations. Interestingly, downsampling resulted in considerably lower sensitivity than the configuration with mixing alone (*t*-test corrected with Bonferroni, *P* < 2e-80), but enhanced specificity compared to configurations without it (*t*-test corrected with Bonferroni, *P* < 5e-30). Moreover, the introduction of leakage atop mixing, had a similar effect to downsampling, albeit to a lower extent. Finally, the difference in F1 scores for both ST-L and ST-D was significantly different from ST-LD (*t*-test corrected with Bonferroni, *P* < 3e-6). However, despite these differences, all configurations tested yielded low F1 scores (Fig. 2C—left) (mean difference from ST-LD ranges between −0.08 and 0.11), suggesting that a property common to all simulations—mixing—is primarily responsible for the results observed. Conceivably, the mixing of different cell types or states can strongly confound DEG detection by masking true DEGs or generating false DEGs depending on the exact mixture present. Thus, even if solutions are found to address leakage and downsampling, spot-level heterogeneity alone appears sufficient to deteriorate both F1 and specificity scores and hamper the accurate detection of DEGs in ST data (Fig. 2C).

### Spot deconvolution combined with classic differentially expressed gene methods does not improve differentially expressed gene recovery

**Figure 3:**
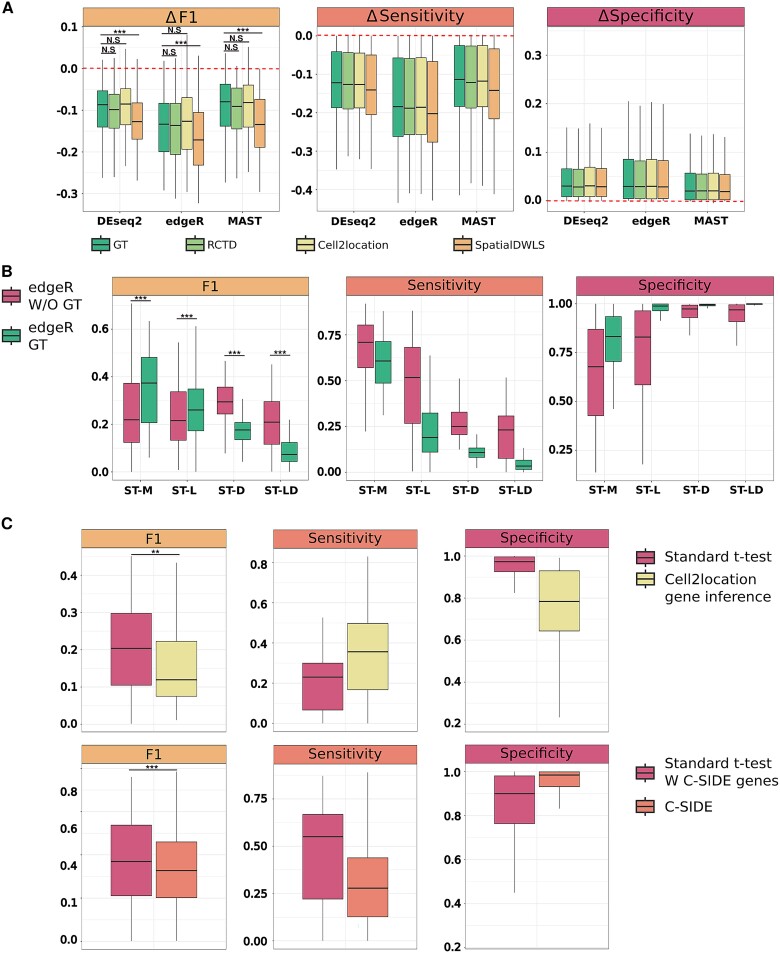
Deconvolution and deconvolution-based inference of cell type–specific expression fails to enhance DEG recovery due to gene expression sparsity. (A) Incorporation of spot-deconvolution results does not boost DEG detection in realistically simulated ST data. F1, sensitivity, and specificity scores derived from DEG tests when the results of different deconvolution methods or the simulated ground truth of cell type ratios were taken into consideration. All scores are expressed as differences (Δ) relative to results obtained from standard, classic DEG approaches. In brief, the model formula for classic DEG algorithms was modified to include cell ratios or abundances as a covariate, while the baseline approach we are comparing to received no additional information about spot composition. Scores above zero represent methods with improved, relative performance compared to the classic DEG approach and vice versa. Of note, only SpatialDWLS had significantly lower F1 scores than the ground truth, but, in all cases, the scores were decreased in comparison to the classic DEG analysis baseline (^*^P ≤ .05, ^*^^*^P ≤ .01, ^*^^*^^*^P ≤ .001). (B) Downsampling results in a significant drop in DEG algorithm performance when deconvolution priors are considered. F1, sensitivity, and specificity scores derived from various spatial configurations that isolate different characteristics of spatial data, computed with (as in Fig. 3A) and without the use of ground-truth cell type ratios. The standard classic DEG method—edgeR—was applied in all scenarios (ST-M, ST with mixing only; ST-L, ST with leakage; ST-D, ST with downsampling; W/O, without additional information; GT, ground truth) (^*^P ≤ .05, ^*^^*^P ≤ .01, ^*^^*^^*^P ≤ .001). (C) Alternative deconvolution-based approaches fail to outperform classic DEG approaches on ST data. F1, sensitivity, and specificity scores derived from the realistic, ST-LD configuration using either a standard *t*-test or cell type–specific deconvolution-based approaches. Top: A cell2location [7] model was used to infer cell type–specific expression and subsequently a *t*-test was applied to the resulting data to detect DEGs. This approach was compared to a standard *t*-test that received no additional information beyond the spots containing the cell types in question. Of note, this analysis was performed on a subset of 160 simulations (one representative example for each DEG % configuration) due to computational limitations. Bottom: C-SIDE-based DEG analysis [23]. As before, this approach was compared to a standard *t*-test that received no additional information beyond the spots containing the cell types in question. Of note, CSIDE returns predictions for only a subset of genes, and an identical subset was used by the standard *t*-test to ensure a fair comparison and all evaluation scores are computed relative to the subset (W, with) (^*^P ≤ .05, ^*^^*^P ≤ .01, ^*^^*^^*^P ≤ .001).

Since spot-level heterogeneity in ST data is a major contributing factor to the loss of DEG algorithm performance, we aimed to investigate whether deconvolution—a process that attempts to resolve heterogeneity by estimating the contribution of each cell type to a spot—could improve results. Several benchmarking papers on spatial deconvolution methods have been published in recent years [8–11], and, based on these, we chose to focus our evaluation on the highest-ranked methods: *RCTD* [6], *cell2location* [7], and *SpatialDWS* [5]. Deconvolution results are typically presented as cell-type ratios per spot or the absolute abundance of a cell type per spot. Thus, to incorporate deconvolution into our pipeline and evaluate whether prior deconvolution of ST data spots could improve DEG detection, we included these metrics as covariates in compatible DEG algorithms (see Methods) when evaluating realistic ST data (ST-LD). In simple terms, this approach controls for differences in cell type composition and seeks to “unmask” true DEGs or correct false DEGs otherwise confounded or created by cellular mixing. Surprisingly, this approach failed to improve DEG recovery (Fig. 3A). Specifically, when comparing the differences in each evaluation metric with and without the inclusion of cell-type ratios or abundances, we observed a consistent decrease in F1 and sensitivity scores when deconvolution priors were included. Moreover, these conclusions held even when we used ground-truth (“GT”) cell ratios as input (see Fig. 3A), suggesting that it is not caused by errors in deconvolution estimates.

Therefore, and as before, to deduce the cause of this discrepancy, we extended our analysis to include all simulation configurations and spatial characteristics in isolation. Notably, the incorporation of deconvolution priors can, as originally anticipated, enhance the results but only in configurations that lack downsampling (i.e. ST-M, ST-L) (*t*-test corrected with Bonferroni, *P* < 2e-6) (Fig. 3B). Downsampling causes a marked deterioration in F1 and sensitivity scores, beyond those observed without deconvolution priors included. Remarkably, a similar trend was observed even when the density of a given cell type was high in both conditions—a state that favors accurate DEG detection (Supp Fig. 4A and B). Application of a less conservative *P*-value threshold (*P* < .01) for DEG detection within this state was able to yield comparable results with and without deconvolution priors (Supp Fig. 4A and C), suggesting that deconvolution may not be a viable solution even under favorable or lenient conditions. We hypothesize that a breakdown in the correlation structure of ST data post-downsampling, specifically due to the introduction of missing values, confounds the ability to accurately estimate gene-to-cell relationships which, in turn, introduces errors when deconvolution priors are considered by DEG algorithms. Regardless of the underlying cause, our observations suggest that while deconvolution can improve classic DEG analysis under certain conditions—or in future technologies with higher sequencing depth—the high sparsity of data, as modeled here by downsampling, severely diminishes this benefit.

### Spot cell–specific expression fails to improve differentially expressed gene detection

Since deconvolution priors were unable to enhance the performance of classic DEG algorithms under realistic conditions, we next sought to test alternative approaches to integrate deconvolved information into the DEG detection pipeline. Specifically, we examined two options: (i) estimation of cell type–specific gene expression using *cell2location* [7], followed by the application of classic DEG methods to the resulting data, and (ii) the use of an ST-specific DEG algorithm, *C-SIDE* [21], that, by design, uses *RCTD* deconvolution results as input. Prediction of cell type–specific expression prior to DEG analysis resulted in significantly lower F1 scores compared to the original analysis that detects DEGs on the data as is (Fig. 3C top, see Methods) (*t*-test *P* = .005), potentially owing to the lack of statistical pooling of information across multiple cell types [24].

Similarly, *C-SIDE* aims to predict cell-specific DEGs along with confidence metrics but only operates on a subset of genes—excluding marker genes used during deconvolution and genes that do not meet specific confidence criteria. As with *cell2location*, the F1 scores obtained by C-SIDE failed to outperform a standard DEG analysis performed without deconvolution but conducted on the same subset of genes selected by C-SIDE (Fig. 3C bottom) (*t*-test *P* = 6e-4). Despite this, we note that, in both cases, the F1 scores are improved compared to our typical results (F1 ≈ 0.4 versus F1 ≈ 0.2) suggesting that while C-SIDE does benefit the process, it is the selection of genes itself that is responsible for this improvement and not other aspects of the algorithm. Taken together and in support of our previous findings, we conclude that the benefits of cell deconvolution methods and the inference of cell-specific expression, in particular, for DEG recovery are diminished by the high sparsity commonly found in real ST data.

### Prior selection of genes can enhance differentially expressed gene detection in spatial data

Since F1 scores were improved by considering only a subset of genes (see Fig. 3C bottom), we speculated that this principle could be expanded upon and that prior selection of genes, based on cell-type specificity, i.e. those genes less likely to be confounded by cellular mixing, could further enhance F1 scores and increase confidence in the results—albeit at the cost of losing a portion of true DEGs present in the data. While cell type–specific genes may still be influenced by the absolute number of cells in a spot, the likelihood of interference from multiple conflicting or additive sources would decrease. Therefore, we decided to implement a gene–cell specificity metric to narrow the gene space and preselect targets with a higher probability of being accurately assessed in ST data.

**Figure 4:**
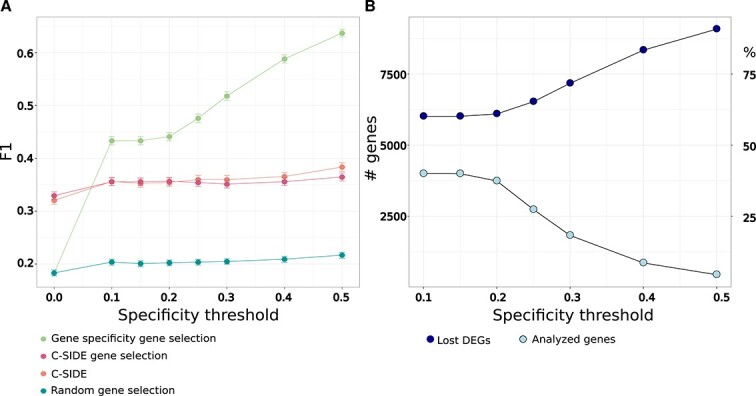
Prior-based selection of genes significantly improves the accurate detection of DEGs in ST data. (A) A novel approach of pre-selecting genes enhances the reliability of DEGs across a wide range of specificity thresholds. F1 scores derived from a standard *t*-test with genes selected by our prior-trained, gene cell-specific thresholds, compared with C-SIDE, a standard *t*-test ran on C-SIDE selected genes, and a standard *t*-test ran on randomly selected genes. The number of genes was kept static between the different approaches to ensure a fair comparison. Each comparison is computed per cell type as before, but all approaches that utilized a standard *t*-test received the entire tissue region that is associated with the condition and not only spots containing the cell type in question (as in Figs 1–3). All F1 scores are relative to the subset of genes tested. (B) Increasing the specificity threshold is a trade-off between DEG reliability and the percentage of DEGs lost. Line plot showing the number of genes passing each threshold and that were analyzed, and a line plot showing the percentage of DEGs lost and which were not evaluated, as a function of the specificity threshold.

We tested two definitions of gene–cell specificity and selected “mean cell specificity,” which yielded the best overall performance (supp Fig. 5A, see Methods). Moreover, we explored varying types of input data. Specifically, in all analyses up until this point, DEGs were assessed per cell type using only the spots containing the cell type of interest—an approach that benefits DEG detection. However, such information is not typically available without further analysis or examination of H&E images. In order to assess the performance of our method under less beneficial but more practical conditions, we simplified our process to compare regions emanating from two conditions regardless of which cell types they contain. Subsequently, we learned gene–cell specificity scores, per gene, based on our simulated SC data (see: Fig. 1A) and assessed whether this selection of genes improves DEG recovery relative to C-SIDE, C-SIDE’s gene selection process in isolation and random gene selection while keeping the preprocessing, input data, and number of genes selected consistent. Remarkably, we observed that, even at the lowest specificity thresholds tested, our gene selection scheme considerably outperforms C-SIDE (*t*-test corrected with Bonferroni, *P* < 3e-11). Moreover, this analysis further indicates that C-SIDE’s advantage is driven, predominantly, by gene selection. Specifically, performance is comparable between C-SIDE (see Fig. 3) and the direct assessment of genes selected by C-SIDE when using a standard DEG test between two regions, where no information about cell types in spots is given (Fig. 4A). Notably, our gene selection method increasingly outperforms the other approaches tested as the threshold increases, suggesting that—as we hypothesized—the higher the gene–cell specificity is, the more reliable the results are. Importantly, both C-SIDE and our prior-based approach have higher F1 scores in comparison to a random selection of genes (*t*-test corrected with Bonferroni, *P* < 4e-62) when any threshold above 0 is applied, emphasizing that the identity of the gene is key and more important than the number of genes selected (Fig. 4A). Of note, the number of genes that are analyzed at a threshold of 0.2—the point at which the trend begins to change—stands at 93.5% of the total, but this value drops dramatically as the threshold increases, while the percentage of DEGs lost increases symmetrically (Fig. 4B). By repeating the same analysis on real scRNA-seq data, which contains 19 cell types and 10 399 genes in total, we observed a similar overall profile to that of our simulated data (Supp Fig. 5B) and likewise a specificity threshold of 0.1—equivalent to 0.2 in simulated data—retained 94.2% of the total genes, demonstrating that our approach may also be applicable to real data despite its size and complexity. At a stricter threshold (0.3), where F1 scores reach >0.5 in our simulations, 1800 genes (45% of the total) remain in the simulated data, while 902 genes (8.6% of the total) remain in the real data, and thus, a biologically relevant number of genes may still be appraised even at strict cutoffs. However, clear benefits are seen throughout the spectrum of specificity values tested. While there is an inherent trade-off in our approach, such as the exclusion of genes and loss of DEGs, our work suggests that genes that are not cell-type specific cannot be reliably detected using current spot-based ST technologies—making this loss largely inconsequential and unavoidable. Overall, we conclude that correct DEG identification can be substantially improved by preselecting genes with high specificity per cell type based on prior knowledge and/or publicly available data.

## Discussion

Here, we investigated, in-depth, the impact of various factors and methodologies on the accuracy of DEG analysis in spot-based ST data. Using a novel simulation platform and extensive simulations, we revealed that accurate DEG detection for realistic ST simulations notably lags behind those of equivalent SC analyses. Surprisingly, we only found marginal differences emanating from the different DEG algorithms tested. Likewise, normalization methods also exhibit minimal effects on the outcome of DEG analysis, suggesting that a preference for raw data utilization may be warranted—as previously proposed in the context of ST data [25]. Among the unique characteristics of ST data, spot heterogeneity—an unavoidable property of spot-based spatial technologies—emerged in our study as the primary contributor to the inability to accurately detect DEGs. While deconvolution algorithms can provide insights about cell type ratios or abundances per spot, our results suggest that, in the presence of high sparsity, their ability to benefit DEG recovery is limited. Moreover, this also applies to data-driven deconvolution-based schemes that infer cell-specific expression from spatial data. While the deconvolution-based algorithm C-SIDE moderately improves F1 scores, our findings suggest that its primary strength lies in gene selection.

Building upon this premise, we introduced a conceptual gene selection scheme that focuses on the specificity of genes per cell type—a scheme that can be applied in an easy and accessible way, without the need for deconvolution or a specific DEG algorithm. The use of cell type–specific genes in DEG analysis has been previously suggested for bulk RNA-seq in combination with deconvolution results to identify the cell types from which the differential expression originated from [26]. Here, we utilize similar information but in a distinct manner. By leveraging the rich cell-expression profiles now available from SC data, we devised a gene–cell specificity scheme and showed that this prior-based approach surpasses the results obtained through data-driven gene selection schemes performed on the experimental data itself. This prior-based approach appears to provide significant benefits even at a low specificity threshold while retaining most of the genes. Additionally, our scheme enables the assessment of marker genes during differential expression analysis, which is not often possible when using deconvolution-based methods such as C-SIDE.

Ultimately, our prior-based approach allows users to flexibly adjust the specificity threshold, balancing the number of genes and the potential DEGs lost with the reliability of the outcome. Importantly, with the explosive growth of SC data and large-scale integration efforts to construct SC atlases and foundation models, building robust priors for cell-specific expression is expected to become easier and ultimately become organism, tissue, and condition specific. Such priors immediately feed into our approach and may be used to further enhance the analysis of ST data. As it stands, we envision that researchers will be able to easily leverage publicly available, precomputed specificity scores, such as those available from cellXgene [27], or compute their own scores from closely matched samples using our formula or others [28], to boost accurate detection of DEGs in their ST data.

It is important to note that the conclusions of this work are limited by the scope of the simulations we ran and that, as of writing, no real-world, gold-standard or ground-truth ST datasets exist to benchmark our approach on. Further exploration of different tissue inputs to the simulation, which affect, for example, the baseline gene expression, leakage coefficients, and the amount of downsampling applied may prove useful. Moreover, our prior-based approach is limited by its inability to determine if a gene is differentially expressed in multiple cell types. However, gene specificity schemes are flexible and may, in the future, incorporate increasingly sophisticated measures to partially overcome this caveat.

In summary, we believe that knowledge integration and building priors for cell-specific expression holds promise as a strategy to enhance DEG analyses, circumventing the need for deconvolution, while significantly simplifying the computation. As new spatial technologies develop, with a richer ability to capture transcriptional depth and SC resolution, the need to leverage cell-specific priors may need re-evaluation, however, with the physical limitations of SC resolution spatial technologies and in contrast, the exponential growth in SC data, the benefits of knowledge-based priors may be here to stay.

## Methods

### Single-cell simulation & data quality control

SC transcriptomes were simulated using the *muscat* (v1.12.0) scRNA-seq simulation platform [29] but with custom modifications. Namely, modifications were made to allow the use of only one reference data source, to ensure less variability in the SC simulation results. As input, an SC reference comprising human distal lung samples from three male donors [30] was used. This dataset was chosen because it contains matched SC and ST data. Nineteen cell types were retained after filtering the data to contain ≥50 cells per type, per donor. Cells were further filtered based on sequencing depth (≥500 counts per cell), cell quality (<12% mitochondrial mRNA), and gene detection rates (>1 count in at least 10 cells, per gene). Finally, genes were removed if they had a very low, mean gene expression. The rationale for this was that the simulation would not be able to simulate such mean expression values accurately, and the log-fold change values of any subsequent DEGs will therefore greatly deviate between the simulation parameters and the actual, calculated values derived from the generated counts. User-configurable parameters for both the SC simulator and the subsequent spatial simulator (below) are generated through a bespoke script and saved as a JavaScript Object Notation (JSON) file. We varied several parameters when generating the simulated SC data, including: the percentage of DEGs in the SC simulation, the random seed used to generate replicates, and the number of total cell types added to the downstream spatial tissue data. An example JSON file, used for our simulations, is available in the linked GitHub repository (see Code and Data Availability), and a list of all tunable simulation parameters can be found in Supplementary Table 1.

### Differentially expressed gene quality control

As the main goal of the simulation pipeline was to establish a rigorous and dependable ground truth for DEG analysis, we ensured that any deviations between the simulated data and the simulation parameters were negligible. Specifically, a set of criteria were applied and tested against. Firstly, the empirical mean expression per gene, per cell type was calculated. If gene means were both zero, the similarity to the simulation parameters could not be tested, and the gene was considered unanalyzable. For a given simulation to pass these criteria, the number of unanalyzed genes was set strictly to ≤5%. Secondly, log-fold changes were calculated by taking the log_2_ of the division between the mean expression of the two conditions. Only an absolute difference of 0.1 in log-fold change between the log-fold change specified in the simulation parameters and the actual one based on simulated data was tolerated. Thirdly, we considered a gene differentially expressed if the *P*-value of a *t*-test between the two conditions’ mean gene expressions was <.01. The difference in the number of DEGs detected and the parameter specified by the user must be <2% for the simulation to be considered valid. Finally, the percentage of the congruent DEGs in the top 100, with the highest log-fold changes, must be ≥60%. Collectively, these latter three conditions were only applied to genes with means above 0 in both conditions, and the simulated dataset had to pass all criteria to be included in the spatial simulator (see below). Thresholds were chosen to balance stringency with the ability to retain a sufficient number of simulated datasets for spatial simulation.

### Spatial simulation

Mixed, Visium-like spots were simulated *in silico* using the *emdann* simulation platform [31], designed to build ST spots from an SC reference. Source code for this package was obtained from: https://github.com/emdann/ST_simulation. Simulated scRNA-seq data (see above) was used as an input. Spatial simulation parameters are loaded from the same JSON file as the SC simulator. Parameters varied between simulation configurations, including the number of cell types, the uniformity of distribution of spots in space, and the density distribution of cells inside a spot. For each cell type, there are two states of uniformity, either a cell type is evenly spread in the tissue or sparsely located, in isolated spots. High uniformity percentages, as can be specified by the user, indicate the likelihood of the cell type to be in the uniform, evenly spread state. Similarly, there are two distinct states for density. A cell type can be either present with high density in spots or with low density in spots, as determined by the user. An example JSON file, which contains the values of these parameters, can be found in the linked GitHub repository (see Code and Data Availability), and a full list of the simulation parameters used can be found in Supplementary Table 1. *Emdann* was customized to support two biological conditions that are mutually exclusive in space so that each spot has cells originating from only one condition—reminiscent of disease samples containing adjacent, healthy tissue segments. Building on the foundation provided by *emdann*, we further introduced the ability to allocate spots to precise locations in 2D space using a real-world spatial reference for the size of the array. To assign spots to a location, we: (i) first chose a random spot in space and then (ii) placed all remaining spots based on random walks of 0, 1, or 2 steps backward or forward in each direction separately. Should the spot be invalid or already occupied, a location from the occupied locations is picked at random, and secondary attempts are made with a maximum of 1000 trials. Once a valid location is found, the random walk continues from that location, and the full selection process is repeated until all the spots are assigned to locations. Tissue shapes typically generated are continuous with a relatively small number of holes. Spots not selected are considered background and contain zero transcripts.

### Transcript leakage

Leakage—that is, the diffusion of transcripts from the source spot to neighboring spots—was simulated and incorporated into our model using the mathematical principles detailed by *SpotClean* [2]. According to their model, the original count in a given spot is the sum of transcripts remaining in that spot along with the transcripts lost and gained due to leakage outwards. Specifically, independent Poisson distributions are used to model the process and the bleeding (i.e. leakage) rate, contamination rate, and a weighted Gaussian kernel are inferred from a separate ST reference. Since in our simulation, original counts are known *a priori*, we used the model to calculate leakage to other spots to obtain ST data with the effects of leakage included. Overall, the counts per simulated spot are the initial transcripts, minus the transcripts that leaked from that spot, plus the gene expression that was received from other spots. Should the leakage from a spot exceed the original count in a spot, a correction is made, and spots that received counts are sampled again with a number equal to the overall leakage. The probability of each spot being chosen is proportional to the difference between the counts that leaked to the spot and the leakage distribution mean, with a minimum of zero.

### Downsampling

Downsampling is performed independently per spot. Specifically, it creates downsampled versions with 10%, 7%, 5%, 3%, 2%, 1%, and 0.5% of the original transcripts. Firstly, the number of transcripts after downsampling per spot is sampled from a normal distribution with a mean equal to the desired percentage from the total. Subsequently, per spot, all genes are sampled together with a probability that is equal to their original proportions in the spot. There is an option to make the downsampling more constrained by ensuring that there are no more counts per gene per spot than in the original count matrix by setting the flag “accurate” to TRUE. This option will take longer to run, and, according to an analysis of a subset of 160 simulations (one representative of each DEG %), it does not affect any DEG results significantly (Supp Fig. 6). An optimal downsample percentage is selected by comparison to the reference data distribution using a Kolmogorov–Smirnov test. The total number of counts per gene and the mean gene expression are calculated, and the percentage that gives the minimal statistic for their sum is chosen.

### Normalization

Simulation data are filtered, such that each gene has at least three counts over all cells, each spot has at least one cell from the SC datas*et* allocated to it, and the number of counts per cell is >0. After invalid spots are removed, genes are filtered again to ensure that all genes have at least 1 count over all cells. The refiltering of genes is predominantly necessary for the configurations that include both leakage and downsampling. The normalization methods that were used are: *SCTransform* (v0.3.5) [32, 33], *DESeq2* (v1.38.2) [34], *scran* (v1.26.1) [35], *loglib*, and *sanity* (commit 81564f7e52d5df47096c083dbf0ce671d1e44801) [36].

*SCTransform* is run with default parameters. For *DESeq2*, a design matrix is built to include both simulated conditions. Since the conditions are mutually exclusive in space, the design formula includes only one of the conditions. As the data are sparse, we calculate geometric means without the zeros prior to size factor calculation. *Scran* runs in two steps, first deconvolving size factors from cell pools and then computing a log-transformed normalized expression matrix based on cell-specific size factors. *Loglib* runs a log transformation of the scaled counts plus a pseudocount to avoid division by zero. *Sanity* runs with default parameters. In order to obtain the normalized count, we used the unnormalized counts and multiplied them by the exponent of the log-transcription quotients.

### Differentially expressed gene analysis

DEG analysis is run on either normalized or unnormalized data. We used a *t*-*test*, *Wilcoxon*, *edgeR* (v3.40.1) [37], *MAST* (v1.24.0) [38], *DESeq2* (v1.38.2) [34], and *C-SIDE* (v2.2.0) [21]. All algorithms calculate DEGs per cell type. Spots taken into account during DEG analysis contain at least one cell from the cell type in question and reside within the tissue boundary. *t*-*test* runs with the default two-sided hypothesis option. If a *t*-*test* runs into an error due to, for example, a low number of samples, the *P*-value is replaced with “NA” (“Not Available”) and is treated as a *P*-value of 1. *Wilcoxon* runs with the default two-sided hypothesis option. *EdgeR* runs with a design matrix including the condition. The estimation of binomial dispersions is done by filtering the total sum of counts above 0. *MAST* runs only on log-transformed data, so, if the normalized data are not log-transformed, a log_2_ with a pseudocount scheme was used. A design matrix with the condition is built per cell type. The zero-inflated regression formula is made up of the proportion of genes detected in each cell and one of the conditions, and the model is tested by a likelihood ratio test of nested models. *Majority* is calculated in the evaluation step, and it treats a gene as a DEG if half + 1 of the other DEG algorithms mentioned above (excluding *C-SIDE*) or more have classified the gene as a DEG. *DESeq2* is the only classic algorithm that does not accept, as input, normalized data. DESeq2 instead ran on filtered data (as described in the Normalization section above). A design matrix with the condition is built per cell type. To avoid zeros in the count matrix when calculating geometric means, we added a pseudo count of 1 to the count matrix and ran the express function *DESeq*. *C-SIDE* first runs *RCTD* in full mode with a cell type threshold of 20 per cell type. This means that for tissues with a low number of spots, some cell types may not have DEG results. Subsequently, we ran *C-SIDE* with a single explanatory variable and used a false discovery rate (FDR) threshold of 0.4 and a weight threshold of 0.8. When comparing *C-SIDE* with a standard DEG algorithm, we chose to use a *t*-*test* since it had similar results to the other DEG algorithms and is both widely used and simplistic.

### Deconvolution analysis

Three state-of-the-art deconvolution algorithms were used: cell2location (v0.1.3) [7], RCTD (spacexr v2.2.0) [6], and SpatialDWLS (Giotto v1.1.2) [5]. Each of the algorithms requires scRNA-seq and spatial data pairs. These were provided by using our simulated scRNA-seq data with cell labels from the simulation in conjunction with the simulated spatial configuration that includes both leakage and downsampling. To ensure the initial conditions to all methods were kept static, we applied the same filtering as described in the Normalization section to all simulated spatial data. Of note, *cell2location* is the only algorithm of the three to require a Python environment.

#### Cell2location

All scRNA-seq models were calculated with a max_epoch of 400 during training and we used 50 cells per location in the spatial model though variation was permitted as per the simulation configuration. Detection_alpha was set to 20, as suggested for data that suffers from technical effects. We lowered the max_epochs to 15000, half of the default amount since we observed that the -ELBO loss function reached a plateau at this value. *RCTD:* RCTD ran using default parameters for full mode, as provided in the GitHub repository of *spacexr*.

#### SpatialDWLS

SpatialDWLS requires the Giotto package and was run according to the published tutorial [39], using default parameters. We applied similar processing steps to the simulated SC data as suggested and used the function *findMarkers_one_vs_all* to detect marker genes that are used as signatures and are essential for the deconvolution process in SpatialDWLS.

### Differentially expressed gene analysis with deconvolution results

In order to use deconvolved information, pertaining to cell-type ratios in spots, in combination with classic DEG algorithms, we used only those DEG algorithms that allow the inclusion of covariates such as *MAST*, *edgeR,* and *DESeq2*. All of the selected algorithms accept a design matrix that can be used to include additional covariates about the experimental design or data. Both *C-SIDE* and *cell2location* were excluded as their functions do not accept cell ratios as covariates since these are estimated from their own models. Similarly, since *ttest* and *wilcoxon* methods do not have an inherent option to account for cell ratios, they were also excluded from testing. In these tests, all spots were included. As before, the design matrix not only specified the condition but also, now, included the cell ratios (for all the spots) and an interaction term between the condition and the cell ratios (condition:cell ratio). This analysis was repeated per cell type since including the full array of cell-type ratios created two unavoidable issues: (i) extremely large covariate matrices, and (ii) the rank of the matrix was not full and would result in convergence errors. In terms of specifics per algorithm, for *edgeR*, the design matrix is as described above but the dispersion is estimated using only the condition and cell ratios. Generalized linear model (GLM) fit tests were performed on the interaction term. In *MAST*, the zero-inflated regression formula contains the condition, cell ratios, and interaction term. The likelihood ratios test is performed with the function *CoefficientHypothesis* on the interaction term. For *DESeq2,* the design formula includes the condition, cell ratios, and interaction term.

### Gene specificity analysis

When devising and benchmarking our own gene-selection method, we examined three inputs for each DEG algorithm : (i) incorporating all spots that contain a specific cell type without the absolute cell quantity or percentage (“containing spots”—as used in the majority of the work); (ii) taking only spots composed predominantly of a specific cell type similar to the output from annotation tools where spots are treated as if they are an SC (“single”); or (iii) encompassing the entire region of a specific condition (“regional”). Ultimately, we chose to use the regional input, as such a scenario is easier to obtain in the real world than “containing spots,” and it was significantly better than the “single” input. Mean specificity was significantly better than both frequency and random specificity (*post hoc* Tukey’s honestly significant difference (HSD) *P*-values 1e-7, and 2e-5, respectively).

Additionally, we assessed two definitions of gene specificity per cell:

(i) the average mean expression scaled by the sum of average mean expression across all cell types (“mean”):


$$ Mean\ specificit{y}_{c_i,g}=\frac{count{s}_{c_i,g}}{\sum_{j=1}^n count{s}_{c_j,g}} $$

where counts = the average mean expression (per cell type), c = cell type, g = gene, and *n* = total number of cell types.

(ii) the frequency of expression, scaled by the sum of frequency across all cell types (“frequency”):


$$ frequency\ specificit{y}_{c_i,g}=\frac{mean\ \left( cell\ count{s}_{c_i,g}>0\right)}{\sum_{j=1}^n mean\left( cell\ count{s}_{c_j,g}>0\right)} $$

where cell counts = a vector of counts of gene *g*, for all cells from cell type *c*, *c* = cell type, *g* = gene, and *n* = total number of cell types.

The decision to use regional input for all classic DEG methods meant that all cell types would have the same *P*-value. We decided that we would only consider the cell type with the highest specificity score since it has the highest chance of being a major contributor to the result. This means that in the evaluation, each gene was evaluated for only one cell type—if at all.

Subsequent to selecting an input datatype and a gene-selection method, we tested the effects of gene specificity on the F1 scores by selecting genes above a certain specificity threshold. Selection was performed after DEG methods ran on the preprocessed count matrix and affected the FDR correction only. We compared a total of four configurations: C-SIDE and standard DEG analysis with genes selected by: prior-based gene specificity (our method), C-SIDE (C-SIDE selected genes only), and randomly selected genes. We matched the number of genes used by C-SIDE to the number of genes that we acquired by applying varying specificity thresholds to ensure a fair comparison. Moreover, we randomly selected the required number of genes out of the C-SIDE subset (without repetitions). If the desired amount was larger than the C-SIDE subset, we used the entire C-SIDE subset and allowed genes to repeat. Standard DEG analyses were performed as follows: *SCTransform* normalization was used with *t*-*test* analysis on all genes, followed by gene selection according to one of the approaches described above, and lastly, *P*-values were corrected according to Benjamini–Hochberg—with the selected number of genes.

### Statistical evaluation

Ground truths are given by the simulation parameters and further validated by robust criteria (see above). If a gene is categorized as non-differentially expressed between the two conditions, it will be treated as such. Throughout the paper, unless stated otherwise, the evaluation per cell type is conducted on all the spots that contain that cell type independently of the percentage of it from the total cells in the spot. Sensitivity is calculated over a few steps, first, by summing all of the genes that are significant according to the DEG algorithm with a threshold of *P* ≤ .01 divided by the number of tested hypotheses according to the Benjamini–Hochberg correction. Subsequently, this sum is divided by the number of true DEGs. The specificity is calculated similarly, but rather, it sums the genes that are classified as non-DEGs and that have a *P*-value over the threshold. The F1 score is a harmonic mean of the sensitivity, also called recall, and the precision. The precision is the ratio between the true positives to all of the positive DEGs identified.

In order to make the comparison to the SC simulations fair, we inflated the number of SCs to be in their thousands, similar to the number of SCs that are contained in an ST simulation. Since the number of cells in each ST simulation is different, we tested configurations between 2000 and 10 000 cells. The configuration with 5000 cells was chosen as it gives maximal F1 scores, along with a 4000 cell configuration that resides at the point where F1 scores begin to decrease (Supp Fig. 7). The SCs are randomly selected from the pool of cells available in order to reach the desired number of cells.

All code was run under R 4.2.2 or Python 3.6.9.

**Key Points**

- Common methods to detect differentially expressed genes (DEGs) do not accurately recover DEGs in data from spatial transcriptomic (ST) spot-based technologies.
- Cellular heterogeneity within spots is a primary contributor to the low performance of these methods.
- The high sparsity of ST data hinders the ability of deconvolution and deconvolution-based methods to enhance DEG recovery.
- Gene selection is a key step in improving DEG reliability in ST data.
- Prior knowledge-based gene cell-type specificity scores significantly enhance the ability to accurately call DEGs in ST data while avoiding the need for complex steps such as deconvolution.

## Supplementary Material

supp_figure_1_bbae621

supp_figure_2_bbae621

supp_figure_3_bbae621

supp_figure_4_bbae621

supp_figure_5_bbae621

supp_figure_6_bbae621

supp_figure_7_bbae621

supp_Figures_legends_bbae621

parameters_table_bbae621

## Data Availability

Code available on GitHub repository https://github.com/shenorrLabTRDF/Spatial_DEG. Filtered SC reference data used in simulations is available at: https://github.com/shenorrLabTRDF/Spatial_DEG/tree/main/simulation/reference_data. Our work made use of the following publicly available datasets: (i) scRNA-seq of human airways (GSE178360); (ii) spatial transcriptomics of human lungs (GSE178361); (iii) spatial transcriptomics of human breast cancer (https://zenodo.org/records/4739739#.YuqFVVpBxkg).
