# Supplementary figures and images for: Cell-specific priors rescue differential gene expression in spatial spot-based technologies
Source: Brief Bioinform. 2024 Dec 16;26(1):bbae621. doi: 10.1093/bib/bbae621 (PMC11647270; doi:10.1093/bib/bbae621)

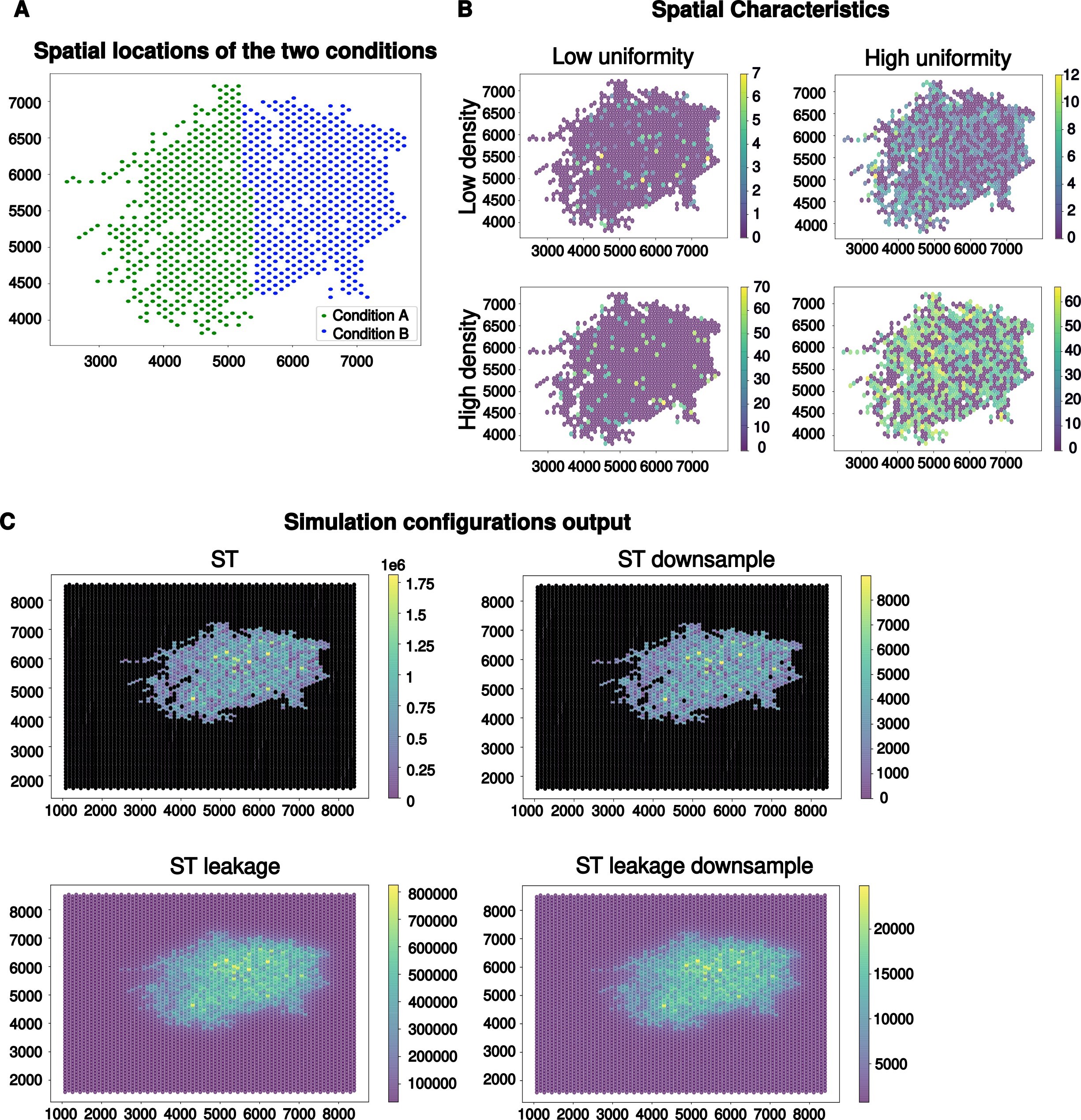

Supplement: supp_figure_1_bbae621 [file supp_figure_1_bbae621.jpeg]

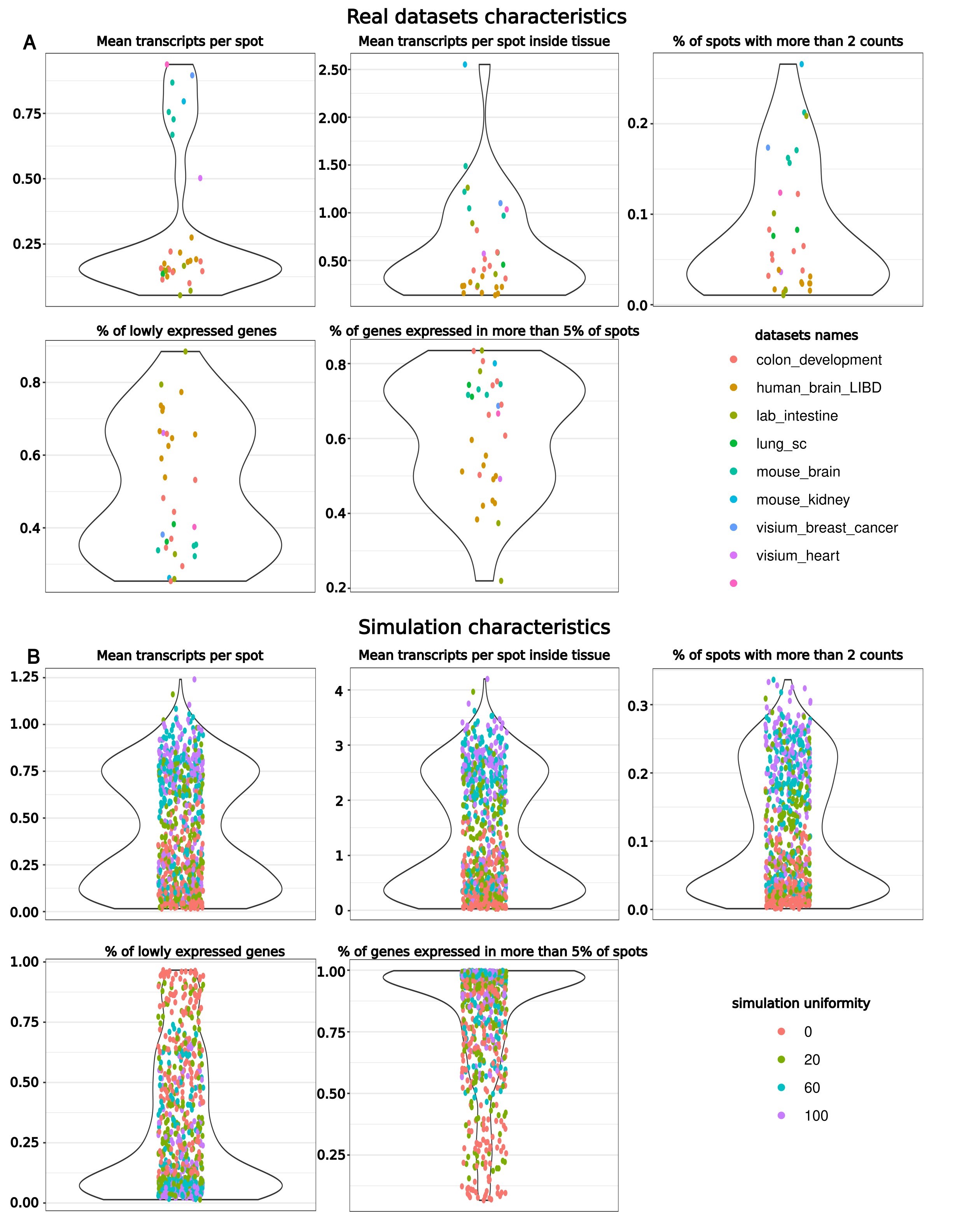

Supplement: supp_figure_2_bbae621 [file supp_figure_2_bbae621.jpeg]

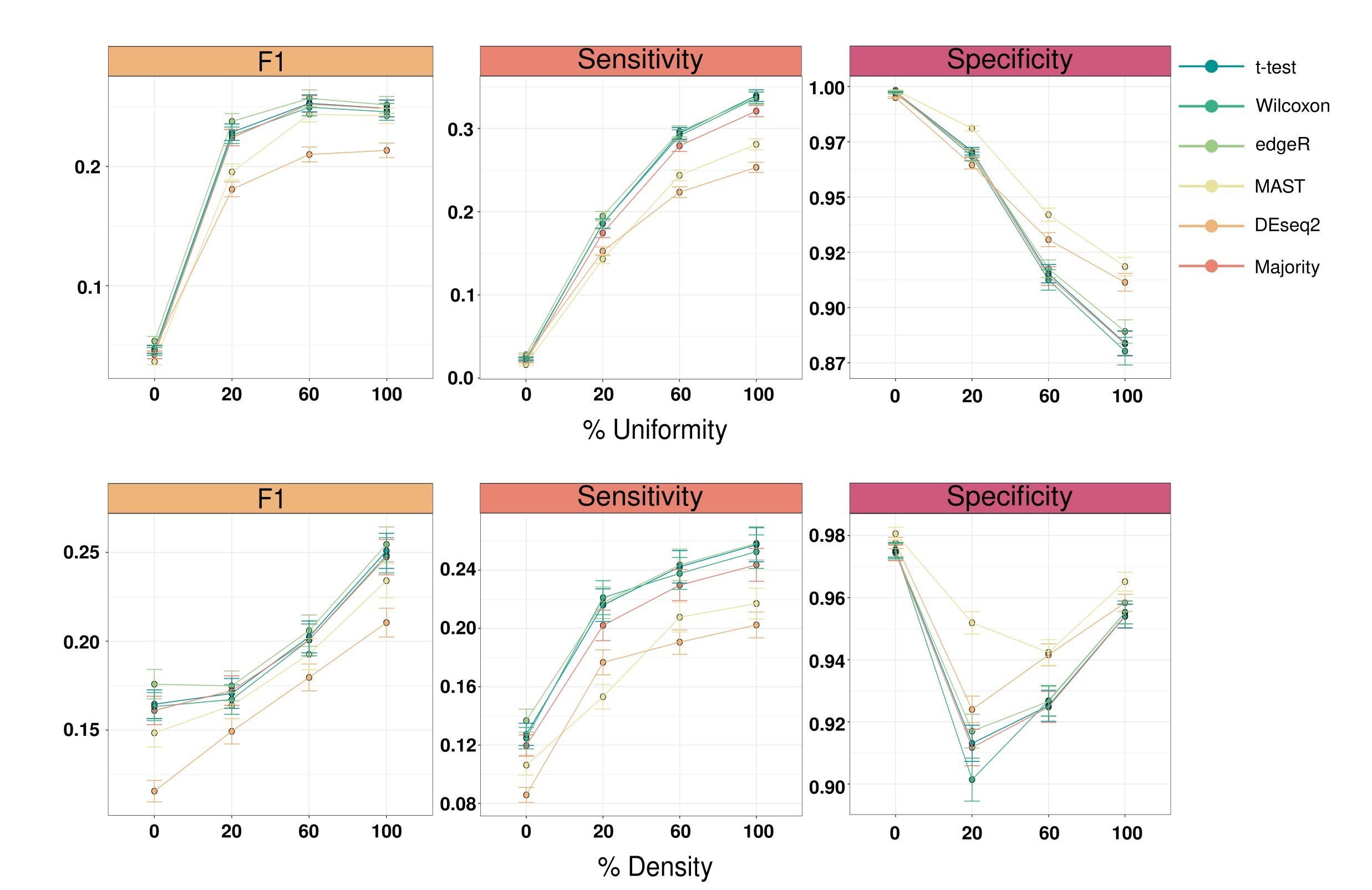

Supplement: supp_figure_3_bbae621 [file supp_figure_3_bbae621.jpeg]

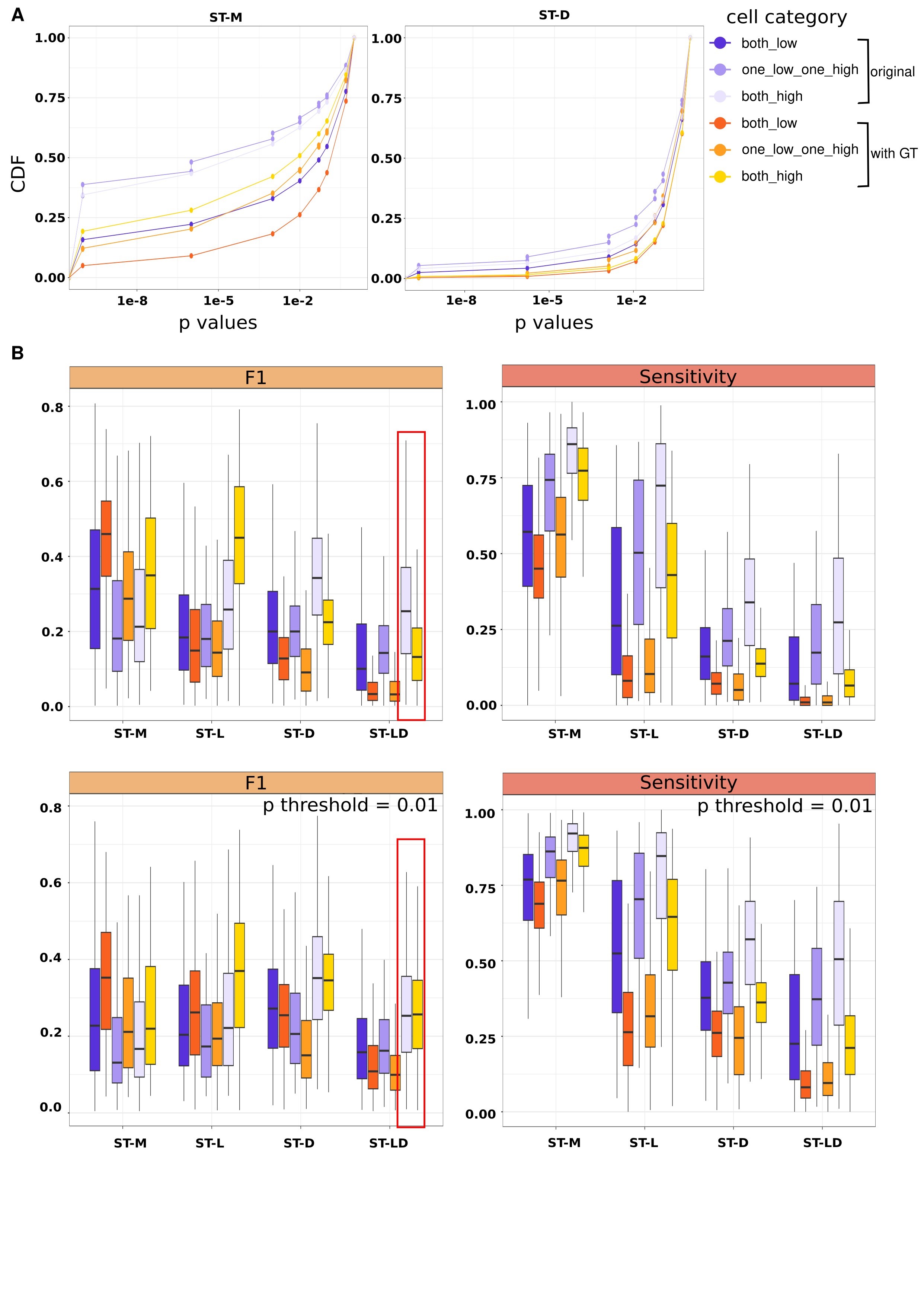

Supplement: supp_figure_4_bbae621 [file supp_figure_4_bbae621.jpeg]

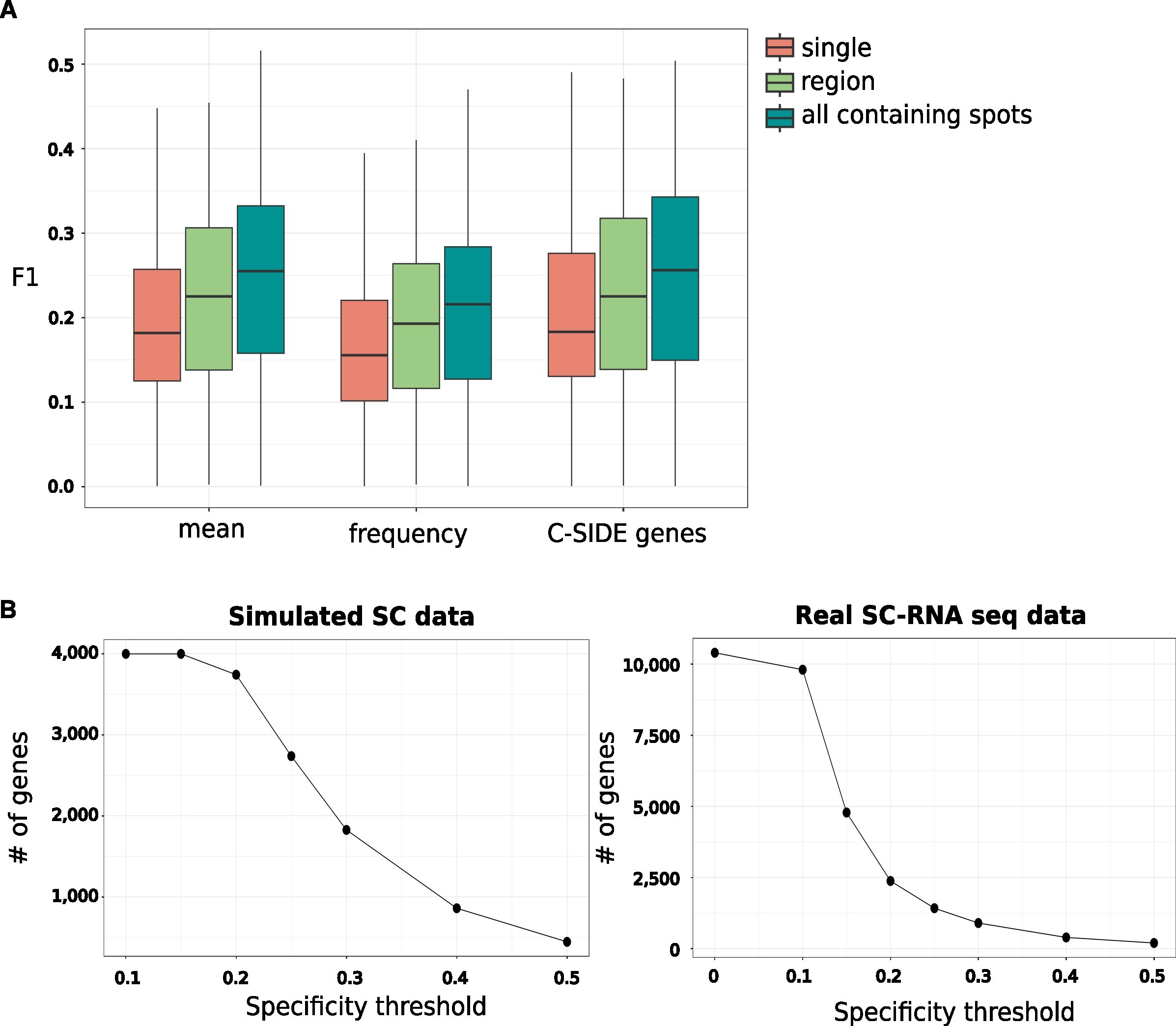

Supplement: supp_figure_5_bbae621 [file supp_figure_5_bbae621.jpeg]

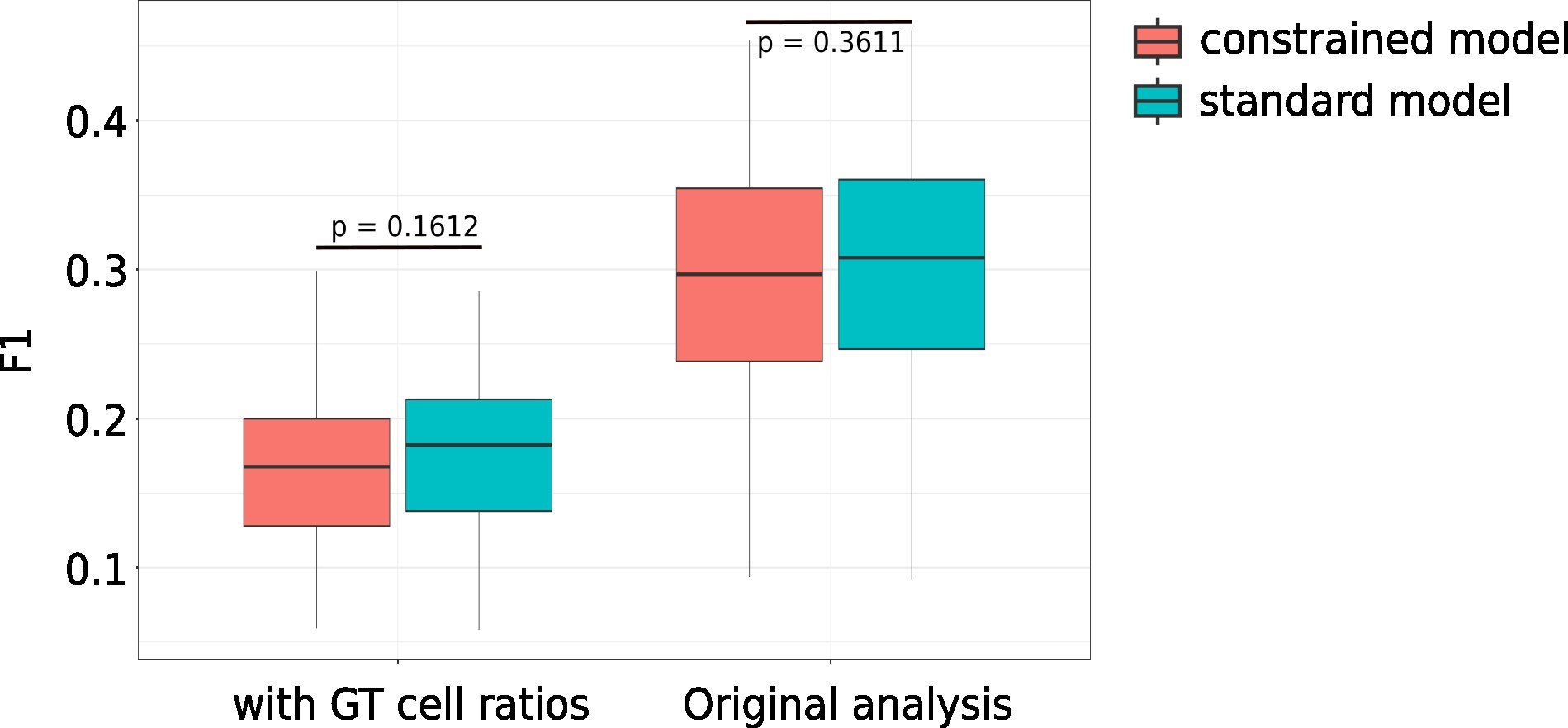

Supplement: supp_figure_6_bbae621 [file supp_figure_6_bbae621.jpeg]

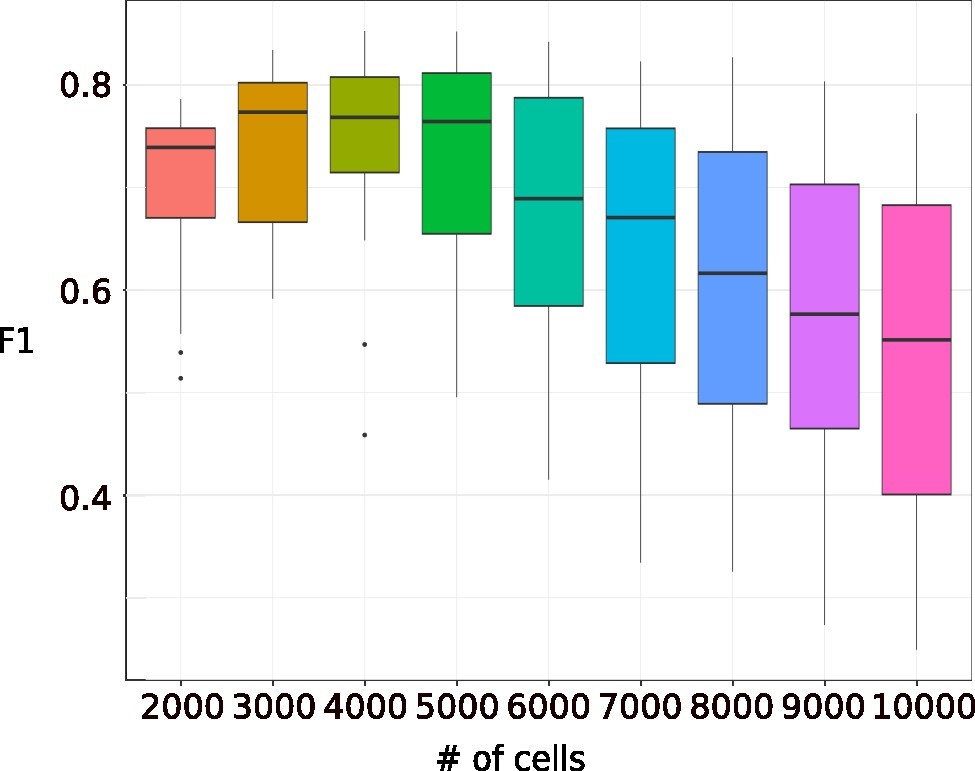

Supplement: supp_figure_7_bbae621 [file supp_figure_7_bbae621.jpeg]
