# Supplementary material for: Cell-specific priors rescue differential gene expression in spatial spot-based technologies
Source: Brief Bioinform. 2024 Dec 16;26(1):bbae621. doi: 10.1093/bib/bbae621 (PMC11647270; doi:10.1093/bib/bbae621)
Supplement: supp_Figures_legends_bbae621 [file supp_figures_legends_bbae621.docx]

**Supplementary Figure 1**

**Spatial simulation output visualizations of unique characteristics and spatial parameters**

**A.** An example of simulated spatial regions comprising of two different simulated conditions, in two dimensional space. Conditions are always mutually exclusive i.e. each spot belongs to only one condition and contains only cells that correspond to that condition.

**B.** The simulation parameters, density and uniformity, are visualized for a single cell type in various combinations: (i) Top - low density; (ii) Bottom - high density; (iii) Left - low uniformity; and (iv) Right - high uniformity. Of note, the scale of counts is significantly lower in the upper row, as expected under conditions of low density. It is possible for any given cell-type to have different spatial characteristics (e.g. uniformity, density) independently between the conditions.

**C.** Different spatial simulation configurations are visualized by their total transcripts per spot. As expected, the configurations without downsampling have a much higher total cell count, while the configurations without leakage have zero transcripts in the background, where no tissue is present.

**Supplementary Figure 2**

**Defining the characteristics of real spatial data**

**A.** Nine different 10X Visium, publicly-available datasets sourced from different organs (colon, brain, intestine, kidney, breast, heart and lymph nodes) and species (mouse and human) were characterized by their: (i) mean transcripts per spot; (ii) mean transcripts per spot when the spot resides inside the tissue; and (iii) the percentage of spots with > 2 counts. The percentage of lowly expressed genes and the expression of genes expressed in more than 5% of the spots was also tested.

**B.** As in (A) but for simulated ST data (the realistic “ST-LD” configuration, with both leakage and downsampling included). Of note, the simulated data range is wider than real data but still encompasses realistic values.

**Supplementary Figure 3**

**The effect of tissue spatial characteristics on the performance of classic DEG algorithms**

F1, sensitivity and specificity scores are described as a function of the uniformity percentage (top) and density percentage (bottom). DEG algorithms are denoted by different colors. Clear trends can be seen that are independent of the DEG method used.

**Supplementary Figure 4**

**Exploration of the performance of ground truth cell type ratios as covariates in classic DEG methods as a function of cell type density in the tissue**

**A.** CDF functions of ST-M and ST-D spatial configurations are shown next to evaluations with and without ground-truth cell type ratios as covariates in *edgeR*. Cell category refers to the cell type densities used under each condition tested. In scenarios where one condition has high density and the other has low density, this data is represented as two points on the graph. Of note, in both configurations, the addition of cell-type ratios inflate the resulting DEG p-values but only in the ST-M case is this beneficial (as reflected by the F1 scores, see supp Fig 4B). Moreover, the differences in p-values in the ST-D configuration are considerably smaller (ST-M - spatial transcriptomics with only mixing , ST-D - spatial transcriptomics with downsampling, GT - ground truth).

**B.** F1 and sensitivity scores of different cell categories with and without ground truth cell type-ratios as covariates in *edgeR*, across different spatial configurations. **Top:** a threshold of 2.5e-6 was used to classify DEGs from non-DEGs. **Bottom:** a more lenient threshold of 0.01 was used to classify DEGs from non-DEGs. Results for the realistic ST-LD configuration, with and without ground truth cell-type ratios, are highlighted (red boxes). Of note, this is the category that is expected to have the highest F1 scores and yet, even in this case, the results are still negatively affected by the inclusion of the cell type-ratios. Importantly, the gap between the bars (with and without cell type-ratios) is reduced when a less strict p-value is applied.

**Supplementary Figure 5**

**Selecting the type of input data for gene-specificity testing and comparing real and simulated specificity thresholding**

**A.** A comparison between different simulated spatial inputs and gene specificity definitions. While using only the spots containing a certain cell type, as was done throughout the majority of the study (Fig. 1-3), benefits DEG detection, it is an input that is not easily accessible. Therefore, we considered two alternative types of input data: (i) treating a spot as a single cell, based on the majority cell-type contained within (“single”); and (ii) using the entire selected region of spots, irrespective of cell type composition (“all containing spots”). Moreover, we tested three different gene specificity definitions (see Methods): (a) genes selected by C-SIDE (“C-SIDE genes”); (b) relative expression of a gene, as calculated by the average mean expression divided by the sum of the expression in all cells (“mean”); and (c) as in (b) but using frequency - that is, the percentage of cells expressing the gene of interest. Both the input configuration and the specificity definition influence the results significantly (two-way ANOVA, p values < 2e-16 for both). Regarding input configurations, the “all containing spots” configuration was considered optimal among those tested, followed by the “regional” configuration, and lastly the “single” configuration (Tukey HSD for containing spots - regional, containing spot - single, regional - single, p values: p=0.003,p <1e-7,p <1e-7,respectively). Of note, the specificity definition based on mean expression (“mean”) had significantly higher F1 scores compared to the definition based on frequencies, but not those selected by C-SIDE (Tukey HSD, p <7e-7, p=0.13 respectively).

**B.** The number of genes analyzed as a function of the specificity threshold. **Left:** Simulated single cell data; a drastic drop in the number of genes per unit increase of the threshold is observed after a threshold of 0.2. **Right:** Real single cell data; a drastic drop is observed after a threshold of 0.1. Overall, a similar trend is observed in both simulated and real data.

**Supplementary Figure 6**

**Comparing the F1 scores derived from downsampling, as applied in the study, with an alternative downsampling scheme.**

F1 scores were compared between the standard model of downsampling applied throughout this study to a more constrained model of downsampling, as calculated using a standard DEG algorithm (*edgeR*) with and without ground truth cell-type ratios as covariates. The constrained model of downsampling ensures that the counts in the downsampled count matrix are never higher than the counts in the original pre-downsampled count matrix, which can occur when the downsampled counts matrix is drawn from a distribution without further constraints. In both cases, the differences in F1 scores are non-significant (p>0.05), and thus either model is valid.

**Supplementary Figure 7**

**The impact of simulated single cell dataset size on F1 scores**

F1 scores as a function of the size of the simulated single cell dataset were evaluated to ensure a fair comparison. Cells from the single cell simulated dataset were sampled and replicated, similarly to ST dataset generation. A dataset size of 5000 cells was selected based on inflection point analysis.
